# Low Glycemic Index Prototype Isomaltulose—Update of Clinical Trials

**DOI:** 10.3390/nu9040381

**Published:** 2017-04-13

**Authors:** Constanze Christin Maresch, Sebastian Friedrich Petry, Stephan Theis, Anja Bosy-Westphal, Thomas Linn

**Affiliations:** 1 Clinical Research Unit, Centre of Internal Medicine, Justus Liebig University, 35392 Giessen, Hesse, Germany; constanze.c.maresch@ernaehrung.uni-giessen.de (C.C.M.); Sebastian.Petry@innere.med.uni-giessen.de (S.F.P.); 2 BENEO-Institute, 67283 Obrigheim/Pfalz, Germany; Stephan.Theis@beneo.com; 3 Applied Nutritional Science/Dietetics, University of Hohenheim, 70599 Stuttgart, Baden-Württemberg, Germany; Anja.Bosy-Westphal@uni-hohenheim.de

**Keywords:** glycemic index, isomaltulose, glucose metabolism, diabetes mellitus, weight-loss maintenance, clinical trials, fertility and pregnancy outcome, sweetened beverages

## Abstract

Low glycemic index diets are supposed to achieve a more beneficial effect on blood glucose control in people with diabetes mellitus and may also provide metabolic benefits for the general population. A prototype of a low-glycemic index carbohydrate is the natural occurring disaccharide isomaltulose that can be commercially produced from sucrose (beet sugar) to industrial scale. It is currently used in various food and drink applications as well as special and clinical nutrition feeds and formula diet as a food ingredient and alternative sugar. Here we provide an overview on clinical trials with isomaltulose including an analysis of its effects on glycemia and fat oxidation as compared to high glycemic index sugars and carbohydrates. In addition, we discuss recent reports on beneficial effects in weight-loss maintenance and pregnancy.

## 1. Carbohydrates with High and Low Glycemic Index on Postprandial Glucose Homeostasis

Plasma glucose levels change according to food supply but are maintained within a narrow range at 5 mmol/L. Glucose homeostasis is the process of maintenance of plasma glucose at a constant concentration (normoglycemia). The rate of glucose entering the circulation and the rate of glucose leaving are tightly regulated. Postprandial glucose excursions are determined by coordinated processes.

After absorption of ingested carbohydrates from the small intestine, glucose is transported to the liver via the portal vein. Simultaneously, the liver converts from production to uptake and storage of glucose [1]. About one-third of the consumed glucose is absorbed by splanchnic tissues while the larger quantity enters the systemic circulation. Systemic glucose load is determined by the rates of intestinal transfer, splanchnic glucose sequestration, and endogenous glucose production [2]. Glucose appears in the circulation already at 15 min after consumption, peaks at about 30 min and decreases gradually thereafter. Synchronous with rising blood glucose levels endogenous glucose production is suppressed [3].

Glycemic index (GI) and glycemic load (GL) are measures to quantitate the level of rising blood glucose by foods [4]. GI is calculated from the incremental area under the postprandial plasma glucose curve of a test food and compared to that following consumption of an equal carbohydrate amount (typically 50 g) from glucose or white bread expressed as percentage of the standard. GL is calculated by multiplying GI with the amount of available carbohydrates in a given food portion or serving size. For example, potatoes at usual serving size provide 30 g of digestible carbohydrates. With a GI of 82% the GL of potatoes is 30 multiplied by 0.82 resulting in 26.2 g.

Plasma glucose levels rise in type 2 diabetic subjects subsequent to a glucose rich drink or more complex meal. The net balance is shifted as the rate of glucose appearance into the blood exceeds the one of removal. Interestingly, absolute rates of glucose disappearance and muscle glucose assimilation are normal or even increased in diabetes mellitus. Using labelled glucose, Wahren and Ekberg demonstrated that hyperglycemia after an oral glucose load resulted first of all from enhanced glucose appearance in plasma [3]. Both failure to adequately regulate endogenous glucose output and reduced splanchnic glucose assimilation could be the causes because appearance of consumed glucose in the circulation was essentially in the same range of people without diabetes mellitus [3].

A type 2 diabetic with fasting blood glucose concentration of 7.8 mmol/L (140 mg/dL) releases as much insulin as a non-diabetic subject. However, when blood glucose exceeds this level and insulin sensitivity decreases, insulin secretion will be progressively exhausted. Consequently, diabetes is associated with elevated fasting but relatively decreased postprandial C-peptide and insulin output.

**Figure 1:**
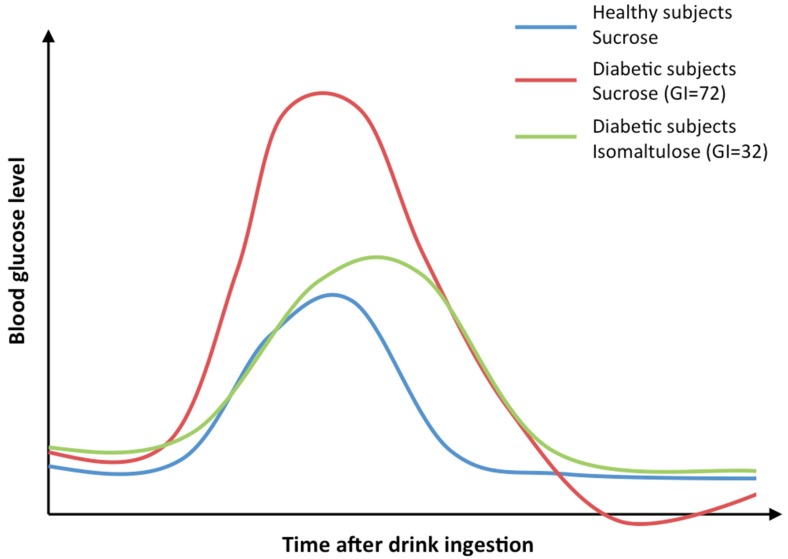
Example curves of a single drink containing 50–75 g carbohydrate of either isomaltulose (Glycemic index GI = 32) or sucrose (GI = 72) [9]. The isomaltulose profile shows nearly 50% reduced peak glucose levels compared to sucrose in diabetic subjects. Moreover, the peak blood glucose of the isomaltulose profile is shifted to the right due to a later time point of the maximum.

Low GI and/or low GL carbohydrates or foods reduce the postprandial glucose rise and are recommended for people with diabetes mellitus (see Figure 1). Starchy staples like pasta or whole-grain breads are low GI foods. Meta-analyses have demonstrated that diets low in GI and GL induce more weight loss and lower glycated hemoglobin than other diets [5,6]. A diet low in carbohydrates was presented as superior to low fat diet and nearly equivalent to Mediterranean diet for weight loss and maintenance [7,8].

In theory, carbohydrates can be partly substituted with other nutrients as glucose is derived from amino acids and fatty acids. However, scientific societies do not recommend abandoning carbohydrates below 40% of total daily calories in people with type 2 diabetes mellitus [10,11]. Therefore, carbohydrates with the benefit of low GI would be preferred over reduction of GL to the low-carb level. Low-digestibility carbohydrates like fibre, resistant starch, or sugar alcohols have a low glycemic response. They are incompletely or not absorbed in the small intestine, but are at least partly fermented by bacteria in the large intestine. There is a small fraction of starch resistant to hydrolysis by amylase, which is found in whole grains and legumes entrapped in a non-digestible matrix.

Given potential health benefits including a reduced caloric content, reduced or no effect on blood glucose levels, and non-cariogenic effect, the prevalence of low-digestible carbohydrates in processed foods is increasing. However, it is controversial in what way low-digestibility carbohydrates and/or low GI foods act mechanistically in diabetes mellitus to balance glucose homeostasis.

## 2. Isomaltulose—Its Manufacturing and Key Characteristics

Isomaltulose, also known by the trade name Palatinose™, is an example of a slow, yet fully digestible carbohydrate with low GI index, which can be administered as a bolus to compare mechanisms of glucose metabolism in humans with various pathological and non-pathological conditions. The chemical denomination of isomaltulose is 6-*O*-α-d-glucopyranosyl-d-fructofuranose with an α-1, 6 glycosidic bond instead of α-1, 2 in its isomer sucrose. In comparison to other alternative low glycemic sugars, such as tagatose or psicose, as well as sugar replacers, such as sugar alcohols (polyols), isomaltulose is unique as it does not escape digestion in the small intestine, and is thus a fully available, yet low-glycemic carbohydrate that provides sustained glucose release. In head-to-head comparison studies with sucrose a number of reports from different groups are available for systematic review (for references see Table 1).

Isomaltulose occurs naturally in small quantities in honey and sugar cane juices [12] and is manufactured on a large scale by enzymatic rearrangement (isomerization) from sucrose (beet sugar). Isomaltulose, the corresponding enzyme for its production (an isomerase), and its source were discovered in the 1950s [12,13]. The α-1, 2-glycosidic bond between the glucose and fructose in sucrose is in an enzymatic step (non-GMO) converted into an α-1, 6-glycosidic bond. This more stable linkage also determines the key physiological characteristic as the maximal velocity value for the hydrolysis of isomaltulose by human small intestinal mucosa homogenate as an enzyme source is only about 26%–45% of sucrose [11].

Isomaltulose has been used as a sugar alternative in foods in Japan and other Asian countries since 1985 [12]. Isomaltulose has been categorized as “generally recognized as safe” (GRAS) in the USA and has been approved following pre-market safety assessment as a food ingredient under the Novel Food regulation in the European Union in 2005 [14] as well as in Australia and New Zealand in 2007. Besides, these countries isomaltulose can be found today in numerous other countries worldwide.

Criteria for characterization and purity, as well as corresponding analytical methods for commercial isomaltulose, are summarized in a monograph of the Food Chemicals Codex (FCC) [15]. Physicochemical properties of isomaltulose allow the substitution of sucrose in existing recipes and processes [12,16]. Isomaltulose has good heat stability upon processing and it is stable in acidic conditions. The sweetness profile of isomaltulose is pure and naturally sucrose-like, and the sweetening power is about half that of sucrose [12,16].

Isomaltulose is used in foods and beverages as a food ingredient and to replace other sugars and maltodextrins. Typical products where isomaltulose is used as ingredient include sports beverages, energy drinks, instant drinks, malt beverages, special and clinical nutrition feeds, breakfast cereals, cereal bars, dairy produce, baked goods, pastry glazings and icings, sugar confectionery (e.g., chocolates, jellies), and more [13,15].

Concerning its physiological properties, isomaltulose is characterized as a fully available, yet slow- and sustained-release carbohydrate. Due to its α-1, 6 glycosidic bond it is digested more slowly than sucrose, which accounts for its lower and slower increases in blood glucose [14]. Isomaltulose is nevertheless completely digestible and available, as was also confirmed in a human ileostomy study [14]. Accordingly, the gastrointestinal tolerance of isomaltulose is good and comparable to sucrose [12,14]. A recent clinical trial conducted in infants aged 4 to 8 months confirmed that this is also the case in this very young age group. Isomaltulose follow-on formula was tolerated and accepted well. No negative effects were found for the number of adverse events, the amount of flatulence, stool consistency, and stool frequency [17].

With a GI value of 32 isomaltulose is characterized as a low GI carbohydrate [9]. In fact, isomaltulose is the only sugar-type carbohydrate which is resistant to oral fermentation (and thus “tooth-friendly”), is a slow-release source, and at the same time is a completely available carbohydrate and glucose supplier (4 kcal/g) [15].

The low/reduced glycemic properties of isomaltulose and its potential to reduce the glycemic response of foods when replacing other sugars (partially or completely) have been confirmed by a positive EFSA opinion [18]. The approval of the corresponding health claim in the European Union has been laid down in the Annex of Regulation EC 432/2012.

In addition, tooth-friendliness has been confirmed by the FDA approval of a corresponding dental health claim in the US Code of Federal Regulations (21CFR §101.80) and by a positive EFSA opinion in the EU [18] with a corresponding claim approved in the Annex of Regulation EC 432/2012 [15].

## 3. Postprandial Blood Glucose and Insulin Levels

We conducted a systematic review for clinical trials that evaluated the application of isomaltulose intake by measurement of blood glucose levels. We searched Pubmed [19], Web of Science [20], ScienceDirect [21], and the Cochrane Library [22] from their inception to 15 December 2016. Search terms included isomaltulose, palatinose, and clinical trials. No language restrictions were imposed. Twenty-seven clinical trials were identified. We decided to focus on studies in adult probands with isomaltulose as beverage as opposed to a solid meal resulting in a total of 12 reports. These studies employed a randomized crossover design throughout but one without randomization procedure [23]. Time of observation was 90–180 min and ingested doses 50–75 g to measure blood glucose and insulin levels every 15 min. A recent report by König et al. investigated fat oxidation in the context of exercise 45 min after drinking bolus [24]. Mean postprandial glucose levels in the first 60 min after ingestion of isomaltulose were 20%–52% lower compared with ingestion of sucrose or maltodextrin [23,25]. Plasma insulin levels and areas under the glucose curve were 30%–50% lower after isomaltulose as opposed to sucrose bolus. This effect was statistically significant in most studies except of two [25,26]. One study [27] reported significantly higher glucagon-like-peptide-1 levels after isomaltulose ingestion, whereas another reported non-significantly higher levels [25].

Two trials assessed subjects with metabolic syndrome and impaired glucose tolerance, respectively. They confirmed reduced postprandial glucose and insulin levels, preferably during the first hour of the isomaltulose load [24,25]. Studies in type 2 diabetic subjects reported on decreased glucose and insulin levels including areas under the curve over 180 min after isomaltulose as compared with sucrose ingestion [23,28,29]. Interestingly, a flattened rise of glucose levels following isomaltulose compared with dextrose was observed also in subjects with type 1 diabetes [30,31]. The subjects were able to reduce normal rapid-acting insulin doses by 50%–75%. Blood lactate levels increased to a greater extent with the ingestion of isomaltulose than with dextrose.

## 4. Glucose Turnover in Type 2 Diabetes

We recently investigated in more detail the effect of isomaltulose ingestion in type 2 diabetic probands to describe the mechanisms involved in the downregulation of postprandial glucose flux [28]. The aim was to follow-up dynamic glucose kinetics at the doses and times utilized in the mentioned clinical studies. For this purpose, the dual-isotope technique was used in combination with the hyperinsulinemic–euglycemic clamp. [6, 6-^2^H_2_]-glucose was intravenously administered to measure the rate of appearance of systemic glucose, and [1-^13^C] labelled isomaltulose was ingested orally to determine the appearance and disappearance of oral glucose. The calculated blood glucose rate of appearance increased on consumption of the disaccharide drink. The maximum of isomaltulose’s blood glucose response was about 60% lower compared with sucrose confirming the observations of the above-mentioned studies. The calculation of the rate of glucose appearance allowed estimation of the absolute amount of glucose in the blood compartment within a given time. We calculated 35% reduced amount of total glucose in the systemic blood circulation after isomaltulose rather than sucrose administration within a time frame of two hours. The rate of oral appearance of glucose also increased postprandially as expected with both disaccharides, but with more than 50% attenuated magnitude of the maximum rate after the isomaltulose load. Follow-up to baseline of ^13^C-labelled isomaltulose indicated that the end of absorption was 50 min later as compared to ^13^C-sucrose. Of the consumed glucose 65% and 92% was found in the systemic circulation after isomaltulose and sucrose intake, respectively. Accordingly, the magnitude of first-pass splanchnic glucose uptake was greater after isomaltulose than after sucrose. This means that more glucose disappeared in the splanchnic organs including the liver rather than in the systemic circulation, which is beneficial for a patient with diabetes mellitus.

Surprisingly, endogenous glucose production decreased significantly to a nadir 105 min after ingestion of isomaltulose. Conversely, after sucrose ingestion, endogenous glucose production increased to a maximum at 60 min. This added up to a 40% lower amount of endogenous glucose being released into the blood stream after isomaltulose than after sucrose intake. The glucose rate of disappearance from blood followed a similar pattern as the rate of appearance.

## 5. Sports Nutrition and Cognitive Performance

The slow-release as well as the low glycemic and low insulinemic properties of isomaltulose have been of particular interest for applications in the area of Sports Nutrition or Cognitive Performance. Consequently, human intervention studies have been conducted on different aspects related to metabolic and associated benefits.

**Figure 2:**
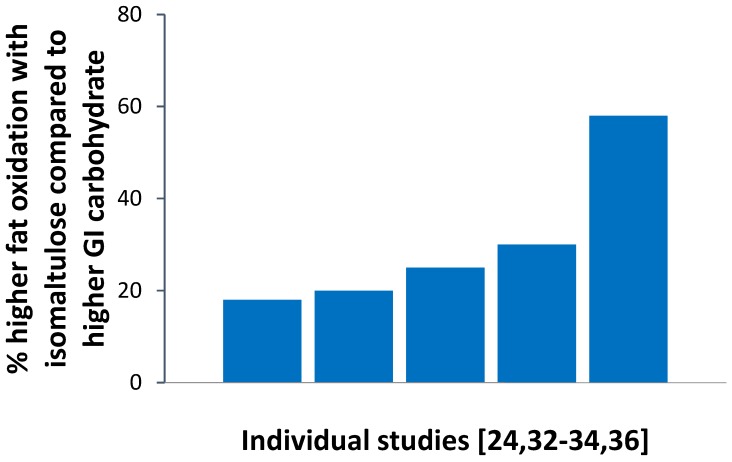
Estimated mean difference in total fat oxidation (% difference between intervention groups) during physical activities between an isomaltulose and a higher glycemic intervention (maltodextrin, glucose, sucrose) in individual studies [24,32,33,34,36]. Mean values were deduced from reported data and graphs of respective publications.

In sports nutrition, isomaltulose is of interest as a slow release source of energy that provides the desired carbohydrate energy for physical activity in a more steady way and at the same time promotes a higher contribution of fat oxidation in energy metabolism than commonly used readily available carbohydrates. This effect is attributed to the lowering effect of isomaltulose on blood glucose and insulin levels compared to readily available high-glycemic carbohydrates. The higher fat oxidation in energy metabolism has been demonstrated during various physical activities in studies including healthy and overweight-to-obese adults, diabetic individuals, as well as trained athletes [24,31,32,33,34,35,36] (see Figure 2).

As regards physical performance outcome with low GI vs. high GI carbohydrates, an improved performance with consumption of low GI carbohydrates was found in some—yet not in all—studies. This mixed finding may be due to different timing and quantities of carbohydrate ingested or the duration, intensity, and type of the exercise protocol [33]. To date, a small number of studies specifically investigated the effects of isomaltulose on performance outcome measures. A recent trial compared the effects of isomaltulose vs. high glycemic maltodextrin ingestion on substrate utilization during endurance exercise and subsequent time trial performance [33]. A more stable blood glucose profile during exercise and higher fat oxidation was obtained with isomaltulose, resulting in better cycling performance in a time trial. These results were explained by the slow-release and low-glycemic properties of isomaltulose which allowed reliance upon more fat oxidation and spare glycogen during the initial exercise period [33]. A small trial with several shortcomings, however, comparing isomaltulose versus a fructose-maltodextrin blend in nine cyclists could not confirm these effects [35]. A double-blinded, counterbalanced, within-group study in resistance-trained men suggests that the addition of isomaltulose as a slow-release carbohydrate and β-hydroxy-β-methylbutyrate to a recovery protein drink may enhance recovery from resistance exercise. Reductions in markers of muscle damage and improved athletic performance were observed [37]. The benefits of isomaltulose on fat oxidation, metabolic control, and incidences of hypoglycemia during physical activity in men with type 1 diabetes mellitus has been the focus of research by the group of Bracken. Isomaltulose consumption during moderate carbohydrate loading before exercise improved glycemic control and protected against hypoglycemia, while maintaining running performance [30,31,36,38].

Furthermore, carbohydrates and their supply of glucose play a central role in cognitive performance and mood [39]. The brain almost exclusively relies on glucose as an energy source and the provision of glucose to the body and brain improves cognitive function compared to placebo (“glucose facilitation effect”). The more sustained and balanced glucose release from isomaltulose is thus of particular interest with respect to beneficial effects in the later phase after a meal.

The potential of isomaltulose in cognitive performance and mood has been specifically examined in studies in healthy children, middle-age adults, and aged adults which compared isomaltulose with higher GI carbohydrates eaten with breakfast [40,41,42,43,44].

A double-blind, repeated-measure study in 75 children aged 5–11 conducted by the department of psychology at Swansea University in Wales, has shown that a breakfast prepared with isomaltulose beneficially influences memory and mood of school children during the morning. Those children who had eaten the isomaltulose breakfast performed significantly better in memory tasks later in the morning than those consuming the higher GI breakfast, both in immediate and delayed memory tests [44]. Moreover, children were in a better mood later in the morning [44].

The effects of breakfasts sweetened with either 40 g isomaltulose, glucose, or sucrose on mood and memory in middle-aged and older adults was examined in a controlled, randomized, double-blind three-arm parallel design study with 155 subjects. It was of particular interest to see whether glucose tolerance of subjects would differentially affect the response to the carbohydrate interventions [43]. This study demonstrated that, contrary to the assumption, subjects with better glucose tolerance benefitted the most from consuming a low-glycemic breakfast. Isomaltulose ingestion resulted in better episodic and working memory as well as better mood. These effects were strongest during the late postprandial period [43]. In contrast, a smaller study with 24 healthy young men could not identify consistent effects in cognitive performance outcomes following single consumption of milk-based drinks containing either isomaltulose (50 g), sucrose (50 g), or a water control (429 mL; 0 Kcal). This was supposed to be related to the combination of experimental design and precise glucoregulation of healthy study subjects [40]. Studies by Taib et al. [42] and Sekartini et al. [41] investigated the effect of different growing-up milks (GUM), including one or several GUMs made up with isomaltulose, on cognitive performance in children aged 5–6 years. Study results indicated that the GUM containing isomaltulose performed best with regard to attention speed, numeric working memory, and delayed episodic memory accuracy [42]. GUMs with different levels of isomaltulose also showed improvements in terms of speed of sustained attention, choice reaction time, new stimuli accuracy, and speed of delayed episodic memory as compared to a higher-glycemic standard GUM [41]. These findings are overall in line with results obtained by Young and Benton [44] as they confirm the beneficial effects of low-glycemic isomaltulose on episodic memory in children. Additionally, they indicate possible favorable effects on measures of working memory and attention.

## 6. Regulation of Body Weight and Composition

A low-GI or low-GL diet may facilitate weight maintenance after weight loss [45,46] especially in insulin sensitive subjects [47]. This is usually explained by the “carbohydrate-insulin theory of obesity”. According to this concept, higher postprandial insulin levels with a high-GI diet promote weight gain by preferentially directing nutrients away from oxidation in muscle towards the synthesis or storage of glucose, lipids, and protein thus shifting diurnal fuel partitioning (the relative storage or breakdown of macronutrients) towards a predominantly anabolic state [48,49]. In line with this hypothesis, studies have shown that enhanced insulin secretion (lower glucose concentrations at the end of an oral glucose tolerance test [50]) and a preferential use of carbohydrate for energy production (i.e., a higher post absorptive RQ) were predictors of weight regain after weight loss [45,51]. On the other hand, studies on the effects of dietary GI on fuel partitioning have largely disagreed [52].

Plausible reasons for the discrepant findings are differences in energy balance and regional insulin sensitivity between studies. The partitioning of nutrients between body tissues is mainly mediated via sensitivity of muscle and adipose tissue to insulin and not only by insulin levels. After caloric restriction, lower insulin-stimulated glucose utilization in skeletal muscles and higher in white adipose tissues contribute to redistribution of glucose from skeletal muscle to adipose tissue thus causing a disproportionate regain in fat mass (for review see [53]). Lower insulin secretion in response to a low glycemic load diet during weight regain may thus partly prevent the disproportionate regain in fat mass [54]. In line with these findings, a high-GI/GL diet after weight loss led to a higher increase in fasting respiratory quotient (RQ) and regain in body weight (in normal weight young men, [45]) or fat mass (in insulin sensitive overweight subjects, [47]) when compared to a low-GI/GL diet. On the other hand, a high-GI/GL diet may facilitate muscle gain in the “post-training window” because insulin sensitivity in skeletal muscle increases after intense exercise or weight training, leading to a higher synthesis of protein and glycogen in muscle cells as opposed to fatty acid storage in adipose tissue. In line with this hypothesis, exercise training has been shown to favor insulin-stimulated glucose uptake in skeletal muscle in contrast to adipose tissue [55]. Conversely, inactivity predominantly impairs insulin sensitivity in skeletal muscle [56] and might thus contribute to shift partitioning towards a higher gain in fat mass. This effect is however dependent on energy balance. At isocaloric conditions, one-week of physical inactivity with high-GI maltodextrin-sucrose sweetened beverages consumption led to higher postprandial and daylong insulin secretion when compared with low-GI isomaltulose sweetened beverages (both 20% energy requirement) whereas whole body fuel selection measured by fasting and postprandial RQ did not differ between both interventions [57]. By contrast, high dietary glycemic load impaired fat oxidation during hypercaloric refeeding but not during caloric restriction [45,58,59,60]. Hyperinsulinemia induced by a high-GI diet may therefore only lead to higher fat storage at a positive energy balance. In addition, it depends on better or maintained insulin sensitivity in adipose tissue when compared to skeletal muscle. This may be the case during weight regain after weight loss, physical inactivity, or in metabolically healthy obese subjects (relative to insulin resistant subjects).

## 7. Pregnancy Outcome

During pregnancy, maternal diet with a high glycemic index (GI) is associated with fetal overgrowth and higher infant body fat mass. A recent meta-analysis of 11 randomized controlled trials involving 1985 women has shown that low-GI diets significantly reduce fasting and 2-h postprandial glucose levels, gestational weight gain, birth weight, and the proportion of babies born large for gestational age [61]. On the other hand, the results for body composition of the infant are less clear. In women at risk of gestational diabetes, a low-GI diet influences offspring birth weight, birth length, and arterial wall thickness in early childhood, but not adiposity or growth trajectory during the first year of life [62]. The results for body composition of the infant may depend on pregnancy stage because of relative changes in infant muscle and adipose tissue insulin sensitivity. In line with this hypothesis, the same group of authors found that, in mid-pregnancy and late-pregnancy, a higher maternal intake of carbohydrate energy or a higher GI was associated with lower infant fat free mass index whereas in late pregnancy higher carbohydrate energy also predicted a lower infant fat mass index [63]. Although there are no long-term studies, both outcomes bear an increased metabolic risk. Insulin-sensitive skeletal muscle is a major recipient of glucose because of its absolute mass in body weight [64]. Impaired development of skeletal muscle may therefore predispose to restricted capacity of glucose homeostasis. Likewise, adipose tissue is the site of safe storage of fat and is indispensable for normal metabolic function. Lack of adipose tissue in early life may potentiate reduced insulin sensitivity in adulthood, however life-long studies will be required to confirm this association. The rationale for such complex and expensive studies can be deduced from a genetic disorder known as lipodystrophy. This condition is associated with severe insulin resistance in affected individuals and might be responsible for ectopic lipid storage in the lean muscle mass [65].

## 8. Conclusions

Low GI diets are recommended in guidelines as they are supposed to achieve a moderate reduction of glycated haemoglobin 1Ac in people with diabetes mellitus with somewhat larger effect when combined with low GL diet [5,64,65] (see Table 1). In this review, we provide an overview on clinical trials with isomaltulose and low-GI diets, analyzing its effects on glucose metabolism and fat oxidation as compared to high GI sugars and carbohydrates. A summary of the main applications for isomaltulose and low-GI diets is given in Table 1.

In summary, low-GI diets and low-GI drinks, based on isomaltulose, were shown to have beneficial effects in various conditions, ranging from clinical use as nutritional treatment of diabetes to improved physical outcome during and after exercise.

In patients with type 2 diabetes, a low-GI diet is recommended to reduce postprandial hyperglycemia and, thereby, improve overall glycemic control. Strikingly, the magnitude of oral and endogenous glucose entering the systemic circulation during the postprandial period after isomaltulose ingestion was significantly lower compared with that after sucrose. Accordingly, 40% less monosaccharides were needed to be disposed from the systemic circulation after ingestion of an isomaltulose drink. However, the same total daily amount of isomaltulose in a mixed diet constituting only approximately 10% of total calories consumed by type 2 diabetic patients over 12 weeks resulted in a reduction of the overall dietary GI of only 6 which was not sufficient to reduce mean blood glucose level as probed by hemoglobin 1Ac [66]. Hence, on a daily basis and within a mixed diet, a higher daily intake level seems to be required to evoke more distinct effects.

Moreover, low glycemic and low insulinemic properties of carbohydrate foods appear of particular interest in Sports Nutrition or Cognitive Performance. In both children, and older and middle-aged adults with good glucose tolerance, lowering the GI and GL of breakfast by the use of isomaltulose improved cognition later in the morning. Consistent in trials with physically active healthy as well as overweight and diabetic individuals, isomaltulose led to a higher fat oxidation in energy metabolism compared with high GI carbohydrates.

With regard to body composition and pregnancy outcome, hyperinsulinemia induced by a high-GI diet may lead to higher fat storage at a positive energy balance. Hyperinsulinemia concomitant with maintained insulin sensitivity in adipose relative to muscle tissue is observed during weight regain after weight loss, physical inactivity, or in metabolically healthy obese subjects relative to insulin resistant subjects and during earlier stages of pregnancy. The effects of dietary interventions in these conditions can only be interpreted on the background of body composition data.

## Figures and Tables

**Table 1:** Applications and observed effects of isomaltulose drinks and low-GI diets.

Application	Observed Effects	Studies Using Isomaltulose Drinks	Studies Using Low-GI Diets
Diabetes mellitus	Isomaltulose drinks: 20%–50% reduced glucose and insulin levels as compared with sucrose or maltodextrin single drink; Delay of peak glucose level; No fermentation up to 75 g per drink; Reduced amount of total glucose in the systemic blood circulation; Increased first-pass splanchnic glucose uptake; Low GI diet: Glycated hemoglobin 1Ac reduced 0.1%–0.5%	[23,24,25,26,27,28,29,30,31]	[5,64,65]
Sports	Promotes a higher contribution of fat oxidation in energy metabolism; Improved physical performance; Protection against hypoglycemia during exercise	[24,31,32,33,34,35,36,40]	[24,37,38]
Cognitive performance	Positive effects on mood; Improved episodic and working memory; Improved attention speed	[41,42]	[43,44]
Body weight and composition	Facilitation of weight maintenance; Increased fat oxidation	[57]	[45,46,47,58,59,60]
Pregnancy outcome	Reduced gestational weight gain and birth weight; Reduced proportion of babies born large for gestational age	-	[61,62,63]
